# Development and validation of a nomogram for predicting postoperative intraluminal hemorrhage in patients undergoing laparoscopic pancreaticoduodenectomy

**DOI:** 10.3389/fsurg.2025.1507434

**Published:** 2025-08-05

**Authors:** Shuai Wang, Dongrui Li, Chengxu Du, Xinda Yang, Lv Haitao

**Affiliations:** Department of Hepatobiliary Surgery, Second Hospital of Hebei Medical University, Shijiazhuang, China

**Keywords:** laparoscopic pancreaticoduodenectomy, periampullary carcinoma, postoperative intraluminal hemorrhage, risk factors, nomogram

## Abstract

**Purpose:**

This study aims to investigate the risk factors for postoperative intraluminal hemorrhage (IPPH) after laparoscopic pancreaticoduodenectomy (LPD), with the aim of enhancing clinical management through the exploration and development of a risk prediction model with those factors.

**Method:**

The clinical data of 326 hospitalized patients between January 2020 and August 2023 who underwent LPD for malignancies were retrospectively selected. The data consisted of general conditions, comorbidities, preoperative treatments, laboratory tests, and postoperative complications. We explored the risk factors associated with postoperative intraluminal hemorrhage using univariate and multivariate logistic regression analyses and developed a predictive model of IPPH after LPD.

**Results:**

The incidence of IPPH in LPD patients was 7.06%. Advanced age (OR = 1.065, 95% CI = 1.001–1.133, *P* = 0.045), low fibrinogen level (OR = 0.485, 95% CI = 0.242–0.972, *P* = 0.041), low albumin level (OR = 0.840, 95% CI = 0.739–0.956, *P* = 0.008), clinically relevant postoperative pancreatic fistula (CR POPF, OR = 4.300, 95% CI = 1.347–13.722, *P* = 0.014), and intra-abdominal infection (IAI, OR = 6.347, 95% CI = 1.454–27.716, *P* = 0.014) were associated with an increased incidence of IPPH. A nomogram was developed and validated with a specificity of 82.2%, a sensitivity of 82.6%, and an AUC value of 0.861 (95% CI 0.783–0.939).

**Conclusion:**

Risk factors for IPPH include advanced age, low fibrinogen levels, low albumin levels, CR POPF, and IAI. These risk factors were used to develop a nomogram for identifying patients at high risk of IPPH, allowing for targeted interventions to address modifiable risk factors promptly and improve patient outcomes.

## 1 Introduction

Periampullary carcinoma is the most aggressive malignancy, including carcinomas located in pancreatic head, distal bile duct, and periampullary duodenal (1, 2). According to reliable statistics, untreated populations have short survival times, averaging less than 18 months (3). Several high-level evidences have indicated that pancreaticoduodenectomy (PD) is currently recommended as the optimal treatment method for periampullary carcinoma, as it provides a complete resection of the tumor margins, thus increasing the possibility of recurrence-free survival for an extended period (1, 4, 5). In recent years, the intensification of the concept of minimally invasive laparoscopic pancreaticoduodenectomy (LPD) has become increasingly popular due to its disadvantages of reduced blood loss, shorter hospital stays, and decreased pain (6–8). Unfortunately, post-pancreatectomy hemorrhage (PPH) negatively affects the efficacy of laparoscopic pancreaticoduodenectomy and causes secondary injuries. These patients may suffer from secondary surgeries and even die (9–11), which poses a severe threat to their survival.

The incidence of PPH ranges from 3% to 16% (12–14). According to the definition provided by the International Study Group of Pancreatic Surgery (ISGPS), PPH is divided into two categories: intraluminal PPH (IPPH) and extraluminal PPH (EPPH) (15). Studies conducted at various institutions have shown that the prevalence of IPPH is steadily increasing compared to EPPH due to advances in surgical approaches (15, 16). IPPH poses challenges in its initial stages because of its elusive detection. In the case of progression to grade C PPH, the associated secondary mortality rate could reach an alarming level 60% (10, 17–19). Numerous factors, including but not limited to age and pancreatic fistula, are related to PPH (10, 17, 19). However, results from contemporary studies showed inconsistency, and the prognostic instruments currently employed in clinical settings demonstrate limited efficacy in anticipating the onset of PPH (13, 20). Currently, there is a lack of research on IPPH. Several studies have shown that surgical factors (e.g., operation time, vascular clamping, C-reactive protein) were risk factors for EPPH and improved surgical procedures to reduce its occurrence (19, 21–26). Nevertheless, it is worth noting that few study investigates the risk factors associated with IPPH.

Given the above conditions, this study analyzed the risk factors for IPPH. Subsequently, these identified factors were employed to develop a prognostic model, enabling the identification of patients with an increased susceptibility to IPPH and ultimately improving their prognosis.

## 2 Materials and methods

### 2.1 Ethical issue

All procedures in the study were performed according to the principles of the Declaration of Helsinki and by the guidelines of Strengthening the Reporting of Surgical Cohort Studies (STROCSS). All patients signed an informed consent form, which was approved by the Ethics Committee of the Second Hospital of Hebei Medical University (2019-R209). To protect the privacy of our patients, all data were anonymized by removing sensitive personal information.

### 2.2 Participants

**Figure 1:**
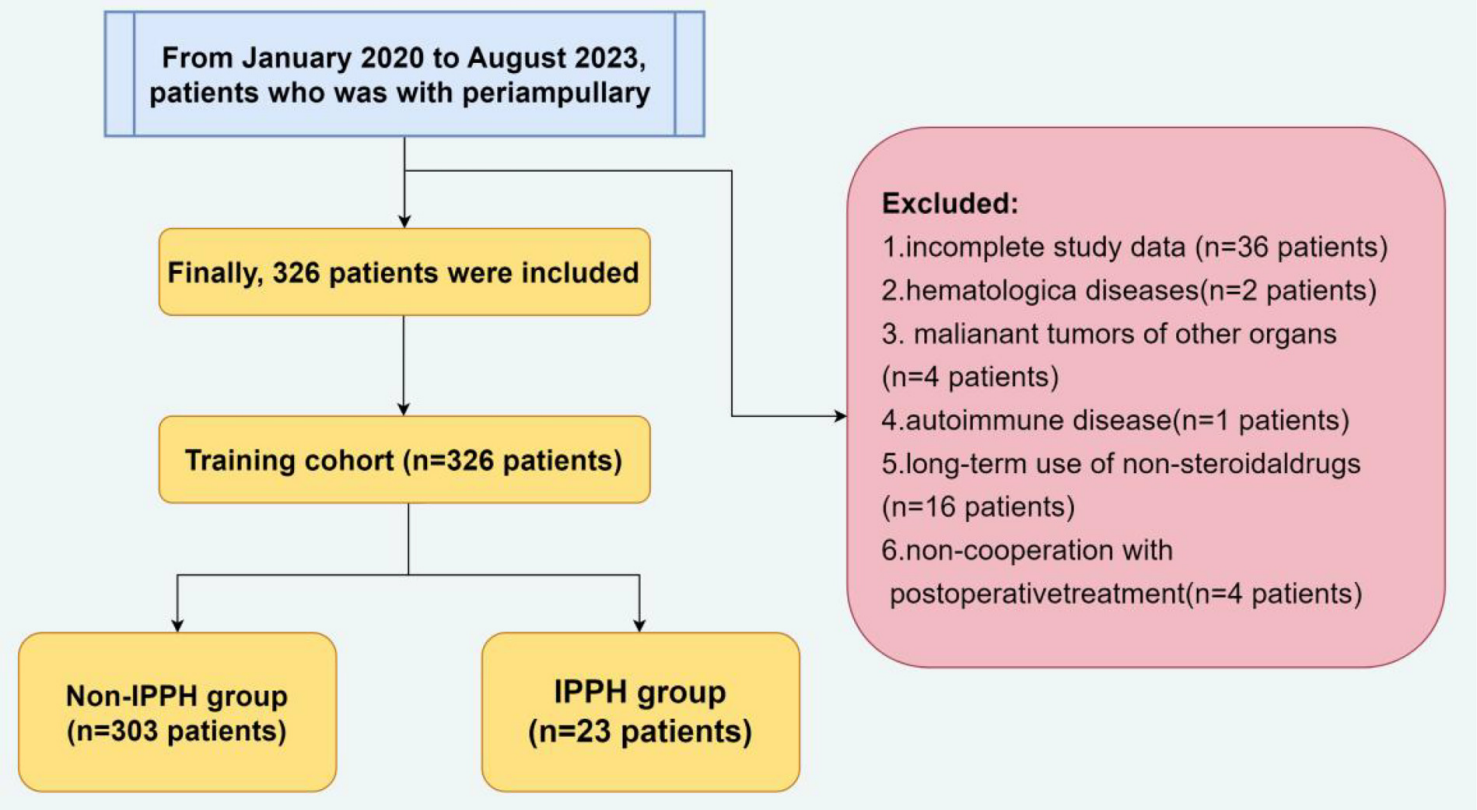
Patients selection flowchart.

A total of 407 hospitalized patients who underwent LPD at the Department of Hepatobiliary Surgery in the Second Hospital of Hebei Medical University between January 2020 and August 2023 for periampullary carcinoma were selected as the objects. The exclusion criteria are: patients with (1) incomplete clinical data; (2) hematological diseases; (3) malignant tumors of other organs; (4) autoimmune disease; (5) long-term use of non-steroidal drugs; (6) non-cooperation with postoperative treatment (Figure 1). This was done to keep the excellent population homogeneous and to allow straightforward interpretation of results.

### 2.3 Data collection

The data included demographics, medical comorbidities, treatment-related, laboratory biomarkers, and postoperative complications. The demographic data includes age, gender (male or female), severe addiction (alcohol or smoking), BMI. Comorbidities included hypertension, diabetes mellitus, coronary heart disease, hepatitis, liver cirrhosis, pancreatitis, previous abdominal surgery. Treatment-related variables included percutaneous transhepatic cholangial drainage (PTCD), blood loss during the surgery, transfusion and operation time. Preoperative laboratory rusults included red blood cell count (RBC), white blood cell count (WBC), platelet count (PLT), albumin (Alb), total bilirubin (TBIL); alanine transaminase (ALT), aspartate transaminase (AST), fibrinogen (FIB), international normalized ratio (INR), creatinine (Cr), prothrombin time (PT). They activated partial thromboplastin time (APTT). Postoperative complications included clinically relevant postoperative pancreatic fistula (CR-POPF), bile leak, intra-abdominal infection (IAI), delayed gastric emptying (DGE), ileusand chyle leak.

ISGPS defined POPF as an amylase level in the abdominal drainage fluid that exceeds three times the upper limit of the average serum value for 3 days or more after surgery. ISGPS further categorized POPF into grades A to C based on the severity of the condition. Grade A represents biochemical fistulae, grade B indicates POPF requiring clinical treatment or intervention, and grade C signifies POPF combined with organ failure or life-threatening situations necessitating surgical intervention. Grades B and C POPF are collectively called clinically relevant POPF (CR-POPF) due to their significant association with prognosis (27). Three days after pancreaticoduodenectomy (PD), the bilirubin concentration in the drainage fluid exceeds three times the concentration in the serum. In such cases, interventional drainage or surgical intervention is necessary to address bile accumulation or diagnose cholestatic peritonitis, indicating a bile leak (28). Intra-abdominal infection (IAI) is characterized by symptoms such as chills, high fever, abdominal distension, and intestinal paralysis, persisting for more than 24 h. Laboratory tests show a significant increase in white blood cell count, hypoproteinemia, and anemia, while imaging studies reveal the presence of fluid in the abdominal cavity. Diagnosis can be confirmed by aspirating pus or detecting bacteria (29). In accordance with the ISGPS, delayed gastric emptying (DGE) is the inability to resume a regular diet by the end of the first week after surgery, including patients requiring prolonged nasogastric tube usage (30). The ISGPS defines chyle leak as the discharge of chyle-like fluid from drains, drain orifices, or wounds occurring on or after the third day following surgery if the concentration of triglycerides exceeds 1.2 mmol/L regardless of the volume (31).

### 2.4 LPD procudures

Pneumoperitoneum was created through a routine 5-hole approach. Subsequently, the operation was divided into two distinct phases: resection and reconstruction. The resection phase included the following steps: (1) performing a Kocher incision, (2) severing the stomach, (3) dissecting the hepatoduodenal ligament, (4) amputating the pancreas, (5) severing the jejunum, (6) dissecting the superior mesenteric vein-portal vein system, and (7) dissecting the superior mesenteric artery-celiac trunk system. The arterial or venous approach selection during surgery is contingent upon the tumor's positioning in relation to the blood vessels. After the resection, a 5-cm specimen was extracted through an incision located beneath the xiphoid process of the upper abdomen, and pneumoperitoneum was reinstated. The digestive tract reconstruction was accomplished using the Child method, which involved implementing various techniques, including the end-to-end mucosal-to-mucosal anastomosis method for pancreatic enterostomy. In this technique, the placement of the pancreatic duct stent was determined by intermittently or continuously suturing it, considering the diameter of the duct. Choledochojejunostomy was performed using an end-to-side anastomosis technique—the anastomosis procedure involved a linear cutting occluder, followed by manual suturing of the joint opening. Two standard abdominal drainage tubes were placed during the surgery. One tube was positioned above the pancreaticojejunostomy and drained through the left abdominal wall, while the other tube was placed below the choledochojejunostomy and drained through the right abdominal wall. No additional drainage methods were employed in this particular case.

### 2.5 Definition

The definition of intraluminal post-pancreatectomy hemorrhage (IPPH) adhered strictly to the guidelines set forth by the International Study Group of Pancreatic Surgery (ISGPS). The ISGPS classification system distinguishes between intraluminal and extraluminal types of hemorrhage based on the location of the bleeding. Within the category of IPPH, various manifestations (e.g., pancreatic surface hemorrhage, peri-anastomotic ulcers, erosions, and biliary hemorrhage) are encompassed (31). The identification of the hemorrhage site was accomplished by gastroscopy or cesarean section.

### 2.6 Statistical analysis

Data were analyzed using SPSS 27.0 (IBM). Measurements were expressed as *x* ± *s*. In normal distribution, the independent sample *t*-test was utilized to compare the two groups, while the rank sum test of two samples was employed for non-normal distribution. Counting data was analyzed using either the *χ*^2^ test or the Fisher exact test. Multivariate logistic regression analysis was performed to identify independent risk factors for predicting IPPH among variables with *P* < 0.10. Subsequently, a nomogram was developed based on these factors using R Programming Language V.4.2.1 (R Foundation). The accuracy, sensitivity and specificity of the nomogram were assessed by the area under the curve (AUC) using receiver operating characteristic (ROC) analysis. Besides, decision curve analysis (DCA) was utilized to investigate the clinical predictive significance of different nomogram variables about the occurrence of IPPH.

## 3 Results

### 3.1 Characteristics of the study patients

A total of 326 patients out of 407 who underwent LPD were deemed eligible for inclusion criteria in this study. Among the enrolled participants, 200 (61.35%) were male, and 126 (38.65%) were female, with a mean age of 59.76 ± 11.24. After LPD, a complication rate of 62.88% (205/326) was observed. Among these patients, 23 were confirmed to have IPPH with a prevalence rate of 7.06% (23/326). IPPH involved the surface of pancreas in 8 (34.78%,8/23), the surface of gastrointestinal anastomosis in 14 (60.87%,14/23), and ulcer of choledochojejunostomy complicated with erosive hemorrhage in 1 (4.34%, 1/23).

### 3.2 Univariate and multivariate analysis

After univariate analysis, seven potential predictors were identified in all variables. Then, this study used multivariate regression analysis to examine the independent risk factors of IPPH (Tables 1, 2). The findings indicate that advanced age (OR = 1.065, 95% CI = 1.001–1.133, *P* = 0.045), low Fib level (OR = 0.485, 95% CI = 0.242–0.972, *P* = 0.041), low Alb level (OR = 0.840, 95% CI = 0.739–0.956), and CR POPF (OR = 4.300, 95% CI = 1.347–13.722) were associated with an increased incidence of IPPH. IAI (OR = 6.347, 95% CI = 1.454–27.716, *P* = 0.014) was also identified as a significant factor. The differences in other factors were not statistically significant (*P* > 0.05).

**Table 1:** Univariate analysis of variables with interest between IPPH and non-IPPH patients.

Variables	Patients without IPPH (*n* = 303)	Patients with IPPH (*n* = 23)	*P*-value
Age	52.93 ± 11.372	66.83 ± 6.00	0.001*
Gender (Female)	118 (38.94%)	8 (34.78%)	0.639
BMI	24.28 ± 3.73	23.69 ± 3.51	0.049*
Alcohol	68 (22.44%)	6 (26.09%)	0.687
Smoking	74 (24.42%)	7 (30.43%)	0.520
Hypertension	110 (36.30%)	7 (30.43%)	0.572
Diabetes mellitus	64 (21.12%)	5 (21.74%)	1.000
Coronary heart disease	23 (7.59%)	3 (13.04%)	0.595
Hepatitis	8 (2.64%)	1 (4.35%)	1.000
Cirrhosis	3 (0.99%)	0	1.000
Pancreatitis	14 (4.62%)	1 (4.35%)	1.000
Abdominal surgery	35 (11.55%)	5 (21.74%)	0.269
PTCD	103 (33.99%)	7 (30.43%)	0.728
Blood loss	571.75 ± 1,034.22	573.91 ± 632.43	0.323
Transfusion	153 (50.50%)	13 (56.52%)	0.577
Operation time	370.85 ± 104.16	360.65 ± 131.49	0.356
CR POPF	32 (10.56%)	8 (34.78%)	0.002*
Bile leak	19 (6.27%)	4 (17.39%)	0.113
IAI	6 (1.98%)	6 (26.09%)	<0.001*
DGE	25 (8.25%)	2 (8.70%)	1.000
Ileus	4 (1.32%)	0	1.000
Chyle leak	2 (0.66%)	0	1.000
RBC	3.98 ± 0.56	3.74 ± 0.66	0.057
WBC	6.29 ± 2.08	6.37 ± 2.26	0.710
Plt	303.99 ± 989.66	253.30 ± 78.67	0.744
Alb	37.94 ± 4.23	34.08 ± 3.76	<0.001*
TBIL	62.76 ± 66.50	73.78 ± 68.35	0.404
ALT	94.49 ± 104.17	80.58 ± 84.20	0.704
AST	71.04 ± 148.41	76.07 ± 90.55	0.689
FIB	3.49 ± 0.81	3.13 ± 0.74	0.018*
INR	1.13 ± 1.68	1.11 ± 0.28	0.209
Cr	61.67 ± 14.41	71.48 ± 46.08	0.575
PT	11.56 ± 1.00	12.30 ± 3.14	0.290
APTT	30.74 ± 3.75	31.32 ± 3.76	0.802

BMI, body mass index; PTCD, percutaneous transhepatic cholangial drainage; CR-POPF, clinically relevant postoperative pancreatic fistula; IAI, Intra-abdominal infection; DGE, delayed gastric emptying; RBC, red blood cell count; WBC, white blood cell count; Plt, platelet count; Alb, albumin; TBIL, total bilirubin; ALT, alanine transaminase; AST, aspartate transaminase, FIB, fibrinogen; INR, international normalized ratio; Cr, creatinine; PT, prothrombin time; APTT, activated partial thromboplastin time;.

* Significant variables.

**Table 2:** Multivariate analyses of the independent risk factors associated with IPPH.

Variables	Odds ratio	95% CI	*P*-value
Alb	0.840	0.739–0.956	0.008*
CR POPF	4.300	1.347–13.722	0.014*
IAI	6.347	1.454–27.716	0.014*
FIB	0.485	0.242–0.972	0.041*
Age	1.065	1.001–1.133	0.045*
BMI	0.887	0.768–1.024	0.103
RBC	0.997	0.403–2.467	0.995

CI, confidential interval; Alb, albumin; CR-POPF, clinically relevant postoperative pancreatic fistula; IAI, Intra-abdominal infection; FIB, fibrinogen; RBC, red blood cell; BMI, Body mass index.

* Significant variables.

### 3.3 Construction and validation

**Figure 2:**
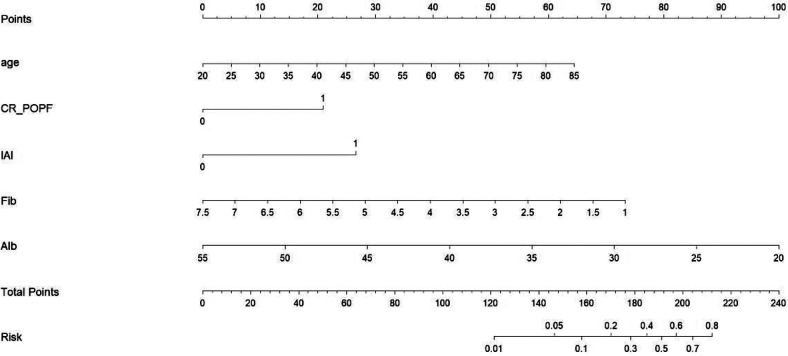
Predictive nomogram for IPPH.

**Figure 3:**
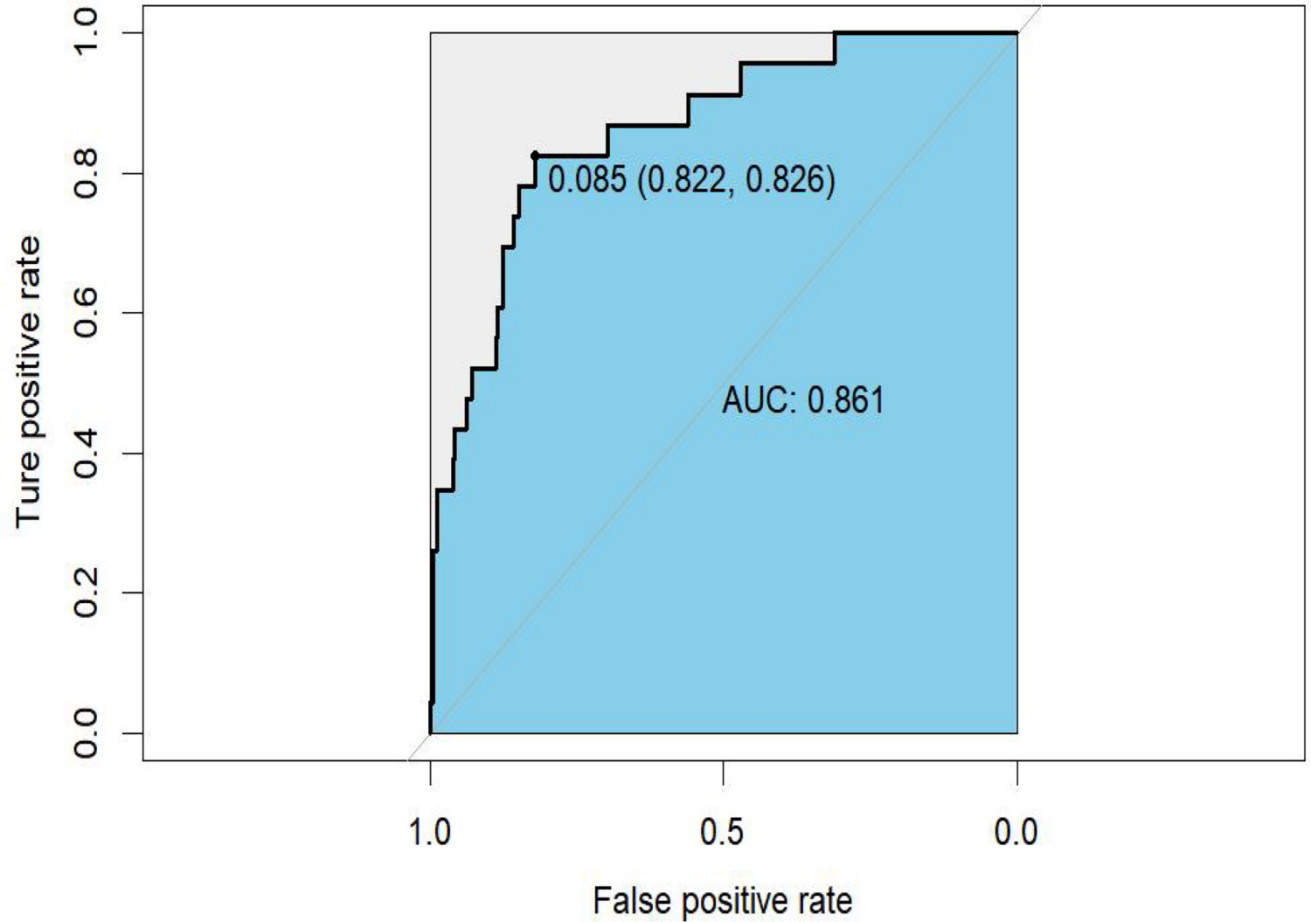
Receiver-operating characteristic (ROC) curves for the nomogram.

The prediction nomogram was constructed based on multivariate analysis findings (Figure 2), offering a more user-friendly and practical instrument for clinical implementation. The area under the curve (AUC) was determined to be 0.861 (95% CI = 0.783–0.939), indicating a substantial degree of sensitivity (82.6%) and specificity (82.2%), thereby attesting to the discriminatory capacity of the model (Figure 3). Furthermore, the c-index and Brier score were computed as 0.862 and 0.052, respectively.

When utilizing the nomogram, the designated variable axis was employed to position the level of each variable. Subsequently, a vertical line was drawn along the axis from the corresponding point to ascertain the risk score. This procedure was iterated for each variable, culminating in the calculation of a cumulative score. The ultimate sum was then located on the “total points” axis, and a vertical line was drawn to intersect the probabilit*y* axis, thereby yielding the projected probability of experiencing IPPH.

### 3.4 Practical significance

**Figure 4:**
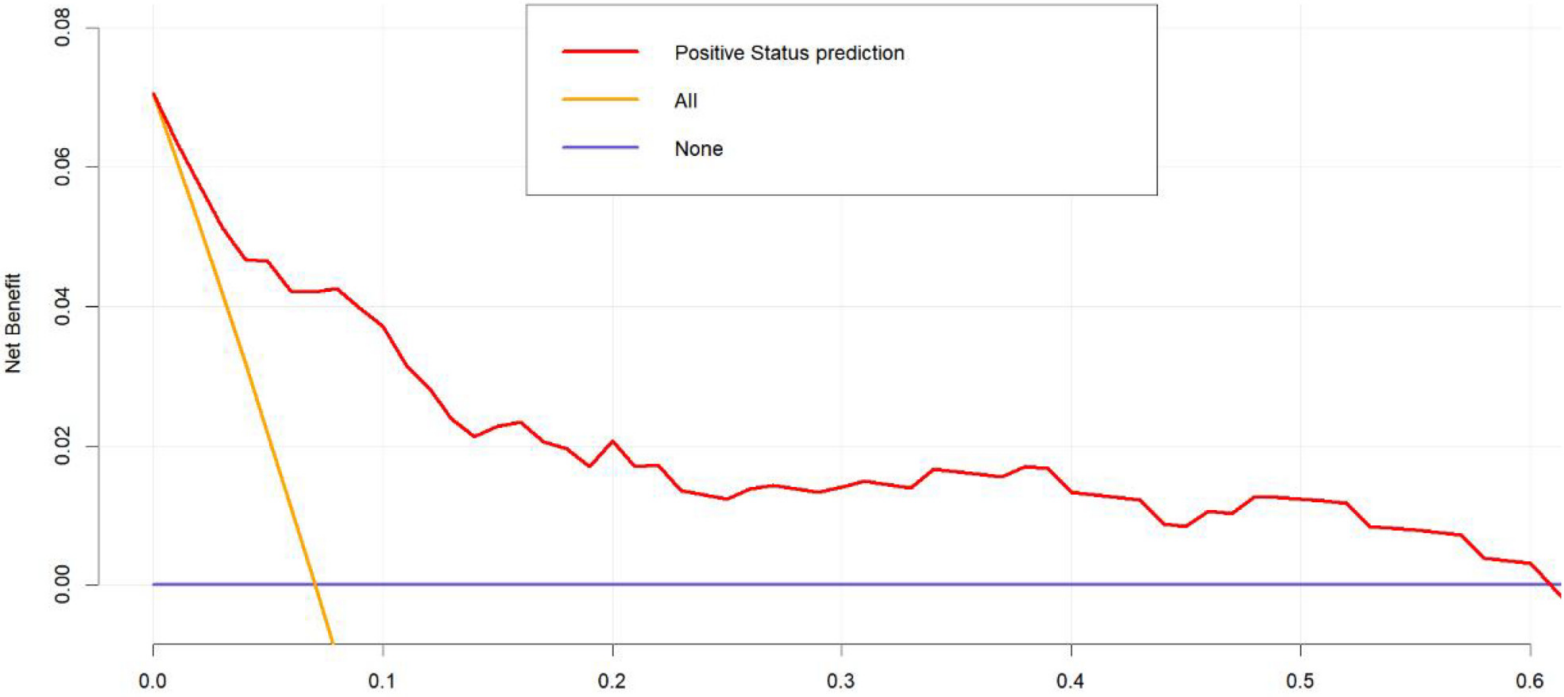
Hosmer-Lemeshow good of fit curves for the nomogram test curves.

**Figure 5:**
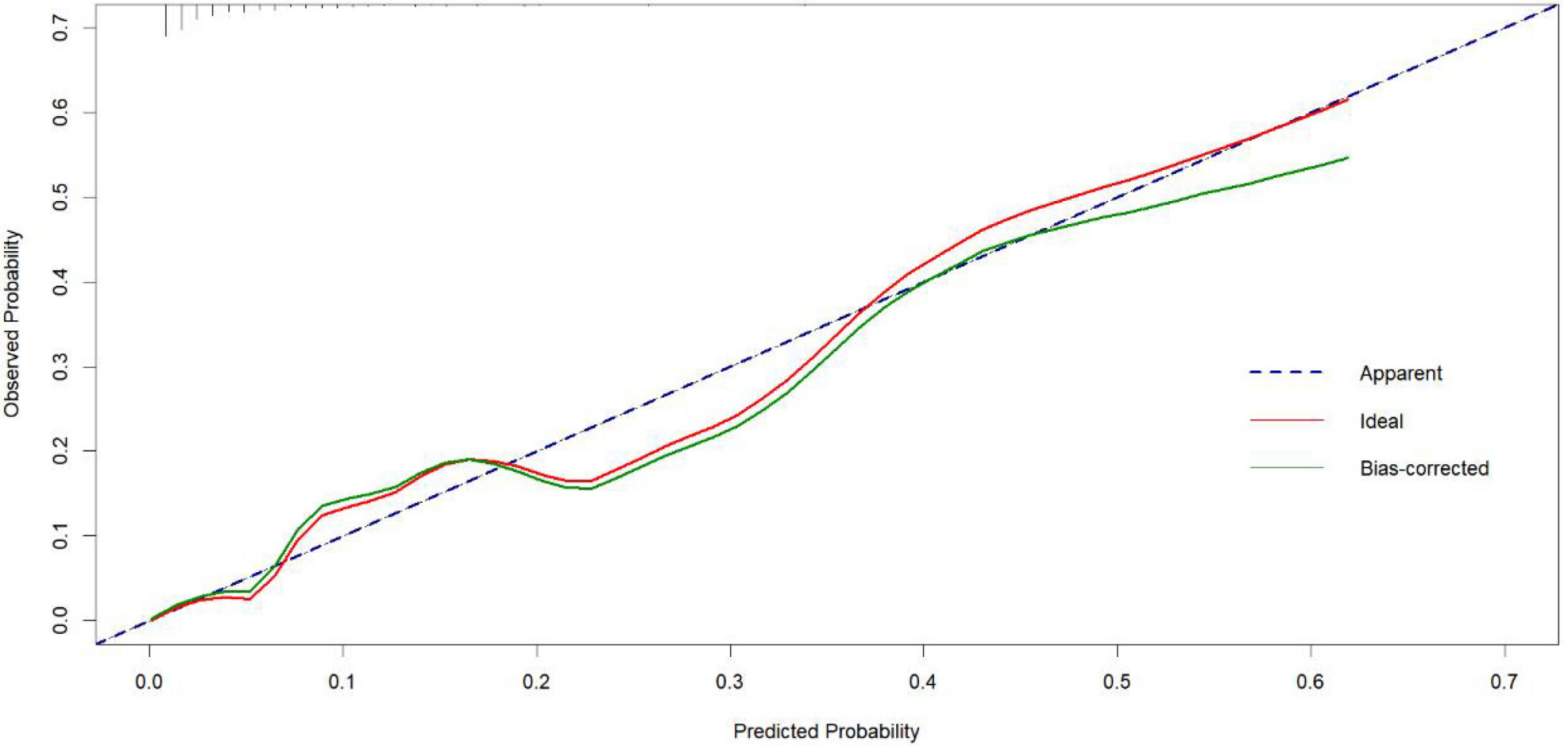
Decision curve analysis for the nomogram test curves.

To validate the net benefit for IPPH of patients who underwent LPD, a decision curve analysis (DCA) and a Hosmer-Lemeshow good of fit test were performed on the prediction model. The Hosmer-Lemeshow X2 statistics of the calibration curve (Figure 4) illustrated a favorable consistency between the probability of predicting postoperative intraluminal hemorrhage and the actual probability of occurrence in patients undergoing LPD. As could be seen in Figure 5, it was clear suggested that the model enhanced the net benefit of the “treat all” or “no treatment” scenario when the threshold probability was between 19% and 100%. The performance of the DCA showed that the model can guide clinical practice well.

## Discussion

The data used in this study were collected by trained investigators who strictly adhere to standardized protocols and regularly update the database based on the patient's condition. This database has proven to be a valuable source of data support in our previous studies. With these data, we demonstrated that the incidence of IPPH was 7.06% (32–34). Advanced age, low Fib level, low Alb level, CR POPF and IAI were identified as independent risk factors for IPPH. We also developed a predictive nomogram model with strong discrimination power and clinical applicability in internal validation. Surgeons can use it to screen immediately on admission and actively optimize risk factors during the perioperative period, providing individualized early intervention if necessary to avoid catastrophic medical consequences in patients with high risk of IPPH.

In the study of risk factors associated with PPH in patients undergoing PD, preoperative elevated C-reactive protein (CRP), increased BMI, higher serum bilirubin levels, elevated international normalized ratio (INR), biliary stent placement, pancreatic duct diameter <0.4 cm, postoperative pancreatic fistula (POPF), and delayed gastric emptying (DGE) were identified as significant risk factors (13, 18, 35). Research on extraluminal PPH (EPPH) has confirmed that younger age, prolonged operative time, and postoperative pancreatic fistula are significant perioperative risk factors for arterial pseudoaneurysm formation (19). These findings differ from our study results, highlighting the complexity of bleeding mechanisms after LPD. Therefore, it is crucial to conduct research by distinguishing between different bleeding sites.

Firstly, regarding age, our study results indicate that advanced age is a significant risk factor for IPPH in patients undergoing LPD. Recent studies by Bingpeng et al. suggest that the short- and long-term outcomes of elderly patients after LPD are comparable to those of younger patients, our findings underscore the importance of considering age as a critical factor in the risk assessment for IPPH (36, 37). However, they also confirmed that elderly LPD patients had a higher frequency of comorbidities compared to younger patients. Another study by Shuichi Aoki, analyzing data from 17,564 patients in the Japanese National Clinical Database, concluded that advanced age is an independent determinant of severe complications following pancreaticoduodenectomy (38).

A research has shown that overweight patients undergoing LPD experience prolonged recovery times and an increased likelihood of developing bile leakage, POPF, and IAI (39). In our study, univariate analysis demonstrated that BMI was statistically significantly lower in patients with IPPH after LPD compared to those without IPPH after LPD (*P* = 0.022). However, binary logistic regression multifactorial analysis indicated that BMI was not an independent risk factor for IPPH after LPD (*P* = 0.214). In summary, BMI cannot be considered a protective factor against intraluminal bleeding following LPD.

This study indicates that Alb and Fib are protective factors against IPPH. Numerous studies have established that preoperative hypoalbuminemia serves as a prognostic indicator for severe postoperative complications in patients undergoing pancreatoduodenectomy (40–45). Although the relationship between fibrinogen levels and the incidence of IPPH has not been extensively studied on a global scale, our research findings indicate that a 1 g/L increase in Fib is associated with a 51.5% decrease in the risk of IPPH following LPD.

A previous research has demonstrated that CR POPF following LPD is lower than open pancreaticoduodenectomy (46, 47). However, the overall incidence of CR POPF remains at 47.3% (48). Researchers have reported that POPF is one of the risk factors for IAI after LPD (49–51). Therefore, there may be an association between CR POPF and IAI, which can impact the prognosis of LPD patients (50). This study indicated both CR POPF and IAI can significantly impede the postoperative recovery of patients. IAI was found to have a more significant effect on IPPH after LPD than CR POPF (18). POPF can lead to alterations in the intra-abdominal microbiota. This disruption triggers a cascade of inflammatory responses, including peri-anastomotic irritation, increased tissue edema, and a higher likelihood of IAI (52). These inflammatory processes further exacerbate the progression of pancreatic fistula (49). Thus, patients experience prolonged postoperative hospitalization and a significant increase in the incidence of IPPH and mortality rate.

Naoya Ikeda et al. demonstrated that obstruction of the portal vein (PV) and superior mesenteric vein (SMV) are risk factors for gastrointestinal bleeding occurring more than 100 days after LPD (53). PV occlusion may be associated with local tumor recurrence or portal hypertension induced by the surgery. Therefore, we need not only perioperative but also longterm follow-up after LPD while taking into IPPH.

There are limitations in this study. First, the single-center design may limit the generalizability of the findings as the results are based on data from a specific population treated at one institution. This could introduce selection bias and affect the applicability of the findings to other settings. In addition, to improve the diagnostic validity of the model, patients with various specific conditions (e.g., hematological diseases, autoimmune disease, or long-term use of non-steroidal drugs) were excluded from this study, and therefore the final results of this study may not be applicable to those specific population.

The potential of artificial intelligence (AI) and machine learning (ML) to advance clinical risk prediction represents a significant avenue for future research endeavors. While our existing model relies on traditional statistical methodologies, AI technologies have exhibited considerable promise in the analysis of high-dimensional clinical datasets and the identification of complex, nonlinear interactions that conventional approaches may overlook. Within the domain of hepatobiliary and pancreatic surgery, recent investigations have highlighted the enhanced accuracy and adaptability of AI-driven models in forecasting postoperative complications, surpassing the performance of traditional methods (54–56). Considering the swift progression and increasing utilization of medical artificial intelligence (AI), especially within China, it is imperative for future research to investigate the incorporation of AI algorithms into predictive models for intraluminal post-pancreatectomy hemorrhage subsequent to laparoscopic pancreaticoduodenectomy. Such methodologies have the potential to enhance the precision of individualized risk stratification, aid in the timely implementation of clinical interventions, and ultimately optimize perioperative outcomes. Although AI was not utilized in the current study, its application signifies a promising direction for the enhancement of surgical risk assessment tools.

## 5 Conclusion

Risk factors for IPPH include advanced age, low fibrinogen levels, low albumin levels, clinically relevant postoperative pancreatic fistula (CR POPF), and intra-abdominal infection (IAI). These risk factors were used to develop a nomogram for identifying patients at high risk of IPPH, allowing for targeted interventions to address modifiable risk factors promptly and improve patient outcomes.

## Data Availability

The original contributions presented in the study are included in the article/Supplementary Material, further inquiries can be directed to the corresponding author.
